# Ectopic mandibular third molar associated with cystic lesion and extraoral approach: case report

**DOI:** 10.1093/jscr/rjae639

**Published:** 2025-09-05

**Authors:** Levi Saulo Rodrigues de Jesus, Bruno Dezen Vieira, Alexander Tadeu Sverzut

**Affiliations:** Postgraduate program in Oral and Maxillofacial Surgery, Faculdade de Odontologia de Piracicaba - Universidade Estadual de Campinas – UNICAMP - Av. Limeira, 901 - Areião, Piracicaba - SP 13414-903, Brasil; Postgraduate program in Oral and Maxillofacial Surgery, Faculdade de Odontologia de Piracicaba - Universidade Estadual de Campinas – UNICAMP - Av. Limeira, 901 - Areião, Piracicaba - SP 13414-903, Brasil; Division of Oral and Maxillofacial Surgery, Faculdade de Odontologia de Piracicaba - Universidade Estadual de Campinas – UNICAMP - Av. Limeira, 901 - Areião, Piracicaba - SP 13414-903, Brasil

**Keywords:** third molar, cystic lesion, maxillofacial, dentistry

## Abstract

Impacted mandibular third molars are classified by its relation with the second molars and with the ascending ramus of the mandible. Ectopic third molars do not obey this classification, being possibly found in the condylar region, in the ascending ramus of the mandible, in the coronoid process or in the base of the mandible. This article aim to present a case of an ectopic mandibular third molar associated with a dentigerous cyst that was removed using a Risdon’s approach. A 49-year-old male patient was referred, after a radiographic finding, for evaluation of the lower right third molar (48) in an ectopic position associated with a radiolucent image, suggestive of cystic lesion, treated with extraoral submandibular approach. This case report can help other maxillofacial surgeons in decision making when they come across with a similar case, reducing the morbidity and improving the surgical results.

## Introduction

The mandibular third molars are the most frequently impacted tooth, affecting 57% of some populations [1], with a higher prevalence in females [2]. The impaction occurs when the tooth has limited space in the mandible or in cases where some hindrance dislocates the third molars from its natural position [3, 4]. Although these teeth were diagnosed incidentally through radiographic findings, they can cause pericoronitis, swelling, recurrent infections, periodontal disease, and a variety of lesions [2].

Impacted mandibular third molars are classified by its relation with the second molars and with the ascending ramus of the mandible [5]. Ectopic third molars do not obey this classification, being possibly found in the condylar region, in the ascending ramus of the mandible, in the coronoid process or in the base of the mandible [2, 4]. When the tooth presents those locations, it can be classified as ectopic [6]. Uncommon and rare regions of the mandible are cited in the literature as ectopic position like the angle of the mandible, ascending ramus, condyle, subcondyle, sigmoid notch, coronoid process, and the border of mandible [7]. This condition can be associated with cystic lesions, which represent the most frequent cause of third molar dislocation, also called secondary ectopy [2, 4, 8].

According to American Association of Oral and Maxillofacial Surgeons, the thirds molars that are associated with disease, or are at risk of developing a disease should be surgically removed. In the absence of disease or significant risk this tooth can be preserved and observed [9]. Despite intraoral approach have limitations and lack of vision it is preferred to be more conservative. On the other hand, extraoral approach have access directly to the tooth, but presents possibility to damage the facial nerve and leave facial scar [2, 4, 7, 8].

The purpose of this article is to present a case of an ectopic mandibular third molar associated with a dentigerous cyst that were removed using a Risdon’s approach.

## Case report

**Figure 1:**
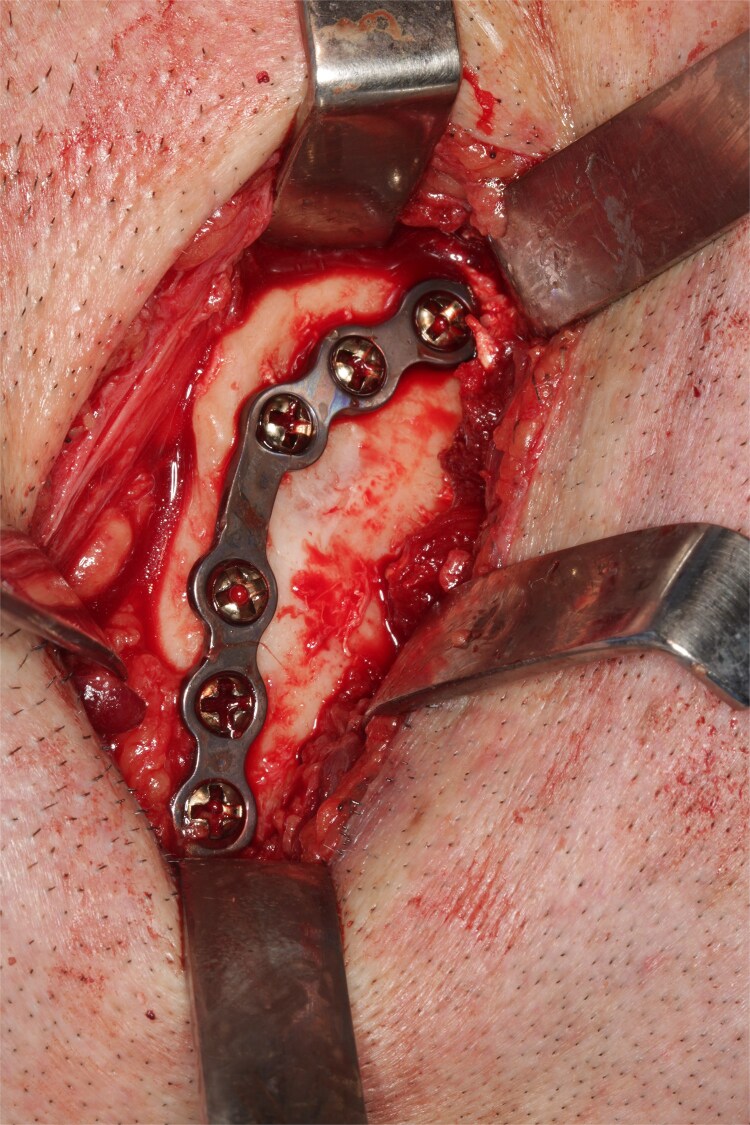
Initial orthopantomography

**Figure 2:**
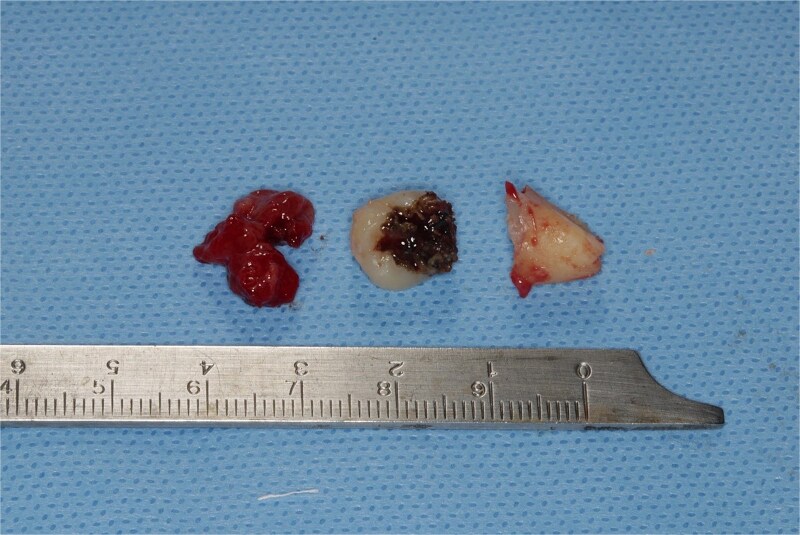
Cone bean CT

**Figure 3:**
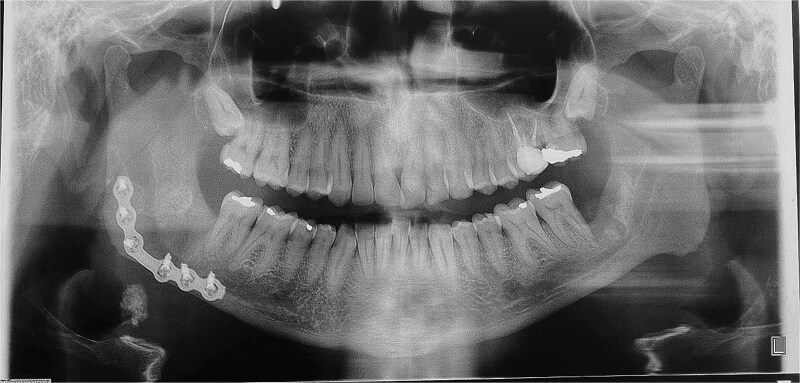
Lesion capsule and sectioned tooth

**Figure 4:**
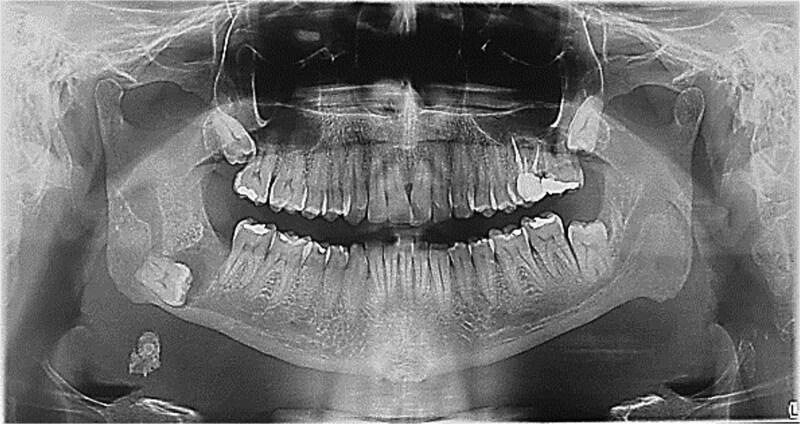
Bone plate 2.4 mm adapted.

**Figure 5:**
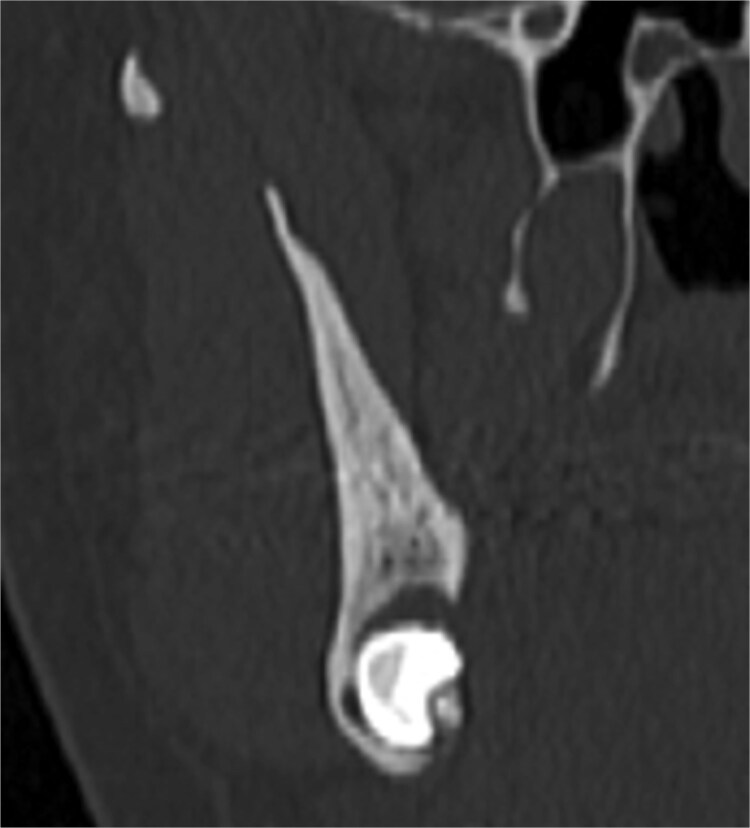
Four months post-operative orthopantomography.

A 49-year-old male patient was referred, after a radiographic finding in a panoramic radiography, for evaluation of the lower right third molar (48) in an ectopic position associated with a radiolucent image, which suggested a cystic lesion (Fig. 1). A cone-beam computed tomography was made. The 3D imaging exams showed the presence of tooth 48 in the mandibular base, in the right angle region, associated with the radiolucent image, lingual fenestration, and intimate relationship between the third molar and the mandibular canal (Fig. 2). Due to the dental position, to have better visualization and less morbidity a submandibular extraoral approach was planned. The tooth removal and cyst curettage were made through the Risdon approach (Fig. 3). A fixation system with reconstruction plate (2.4 mm) was used to prevent a mandibular pathological fracture (Fig. 4). Anatomopathological examination of the cystic capsule was performed with a diagnosis of dentigerous cyst. In the postoperative period, the patient reported alteration in the sensitivity of the inferior alveolar nerve. Postoperative radiographic examinations showed adequate adaptation of the fixation system and complete surgical removal of the tooth and associated lesion. Orthopantomography with 4 months follow-up showed bone repair and adequate adaptation of the fixation system (Fig. 5).

## Discussion

The ectopic third molar usually is asymptomatic and difficult to identify. However, the clinical symptoms include swelling, limited mouth opening, pain, discharging fistula, or temporomandibular joint discomfort [4, 6, 10]. The diagnosis of ectopic third molar is completely formed with radiographic findings such as panoramic radiograph or computerized tomography, that performs 3-dimensional information and precise location of the ectopic tooth and surrounding structures [5, 11]. The panoramic radiography showed the location of the third molar in the present case, but only after the cone-beam tomography examination the treatment plan was defined. The lingual position, the close relationship with the mandibular canal and the caudal position determined the removal through the submandibular approach.

Ectopic tooth is defined as located in a non-physiologic area like mandibular condyle, orbit, coronoid process, nasal cavity, nasal septum, palate chin, and maxillary antrum [3, 7] In the case presented, the mandibular third molar was located in the base of the mandible, as the showed for some studies [2, 6, 12, 13]. Those studies also showed cases where the mandibular molar was associated with a radiolucent image, which suggest a lesion, as the present case.

The etiology is not clear, but suggests that this rare condition is caused for, deviant position of tooth germ, aberrant eruption pattern, and trauma or displaced by pathological lesions such as cyst or tumor in the jaw [7]. In the presented case, the inferior third molar was associated with an infected dentigerous cyst. This is a finding showed in other studies in the literature [2, 3, 6, 12, 13], being an indications for ectopic third molar’s removal [10].

Selection of surgical approach is linked to the location of tooth, surgical morbidity and surgeon experience, most patients can be treated in an intraoral approach whenever possible [7]. However, intraoral approach can’t provide adequate surgical field and clear visualization for some anatomical area or in patients with severe restriction of mouth opening [14, 15], that was the case in the presented patient. The intraoral approach wouldn’t give adequate visualization. Besides this, an extensive osteotomy would be necessary to expose the tooth and the lingual position of the third molar would require a lingual flap that could cause lingual nerve damage.

Ectopic third molar in the base of the mandible should be extracted with an extraoral approach by submandibular or retromandibular incision, to get good surgical exposure [4]. A Risdon’s approach was selected to minimize the risks of lingual paresthesia, excessive bone loss due to the osteotomy and consequent mandible’s fracture. The submandibular approach also was the chosen option for the installation of the fixation system. Other studies used fixation system after the ectopic molars removal’s [10, 12, 13]. A load-bearing system was required due the lack of bone that remained in the mandible’s body.

## Conclusion

The present study shows an unusual ectopic third molar located in the base of the mandible with a dentigerous cyst that was removed using an extraoral approach. The relate of this case can help other maxillofacial surgeons in decision making when they come across with a similar case, reducing the morbidity and improving the surgical results.
